# Tunneling-induced Talbot effect

**DOI:** 10.1038/s41598-021-86289-w

**Published:** 2021-03-25

**Authors:** Babak Azizi, Zahra Amini Sabegh, Mohammad Mahmoudi, Saifollah Rasouli

**Affiliations:** 1 Department of Physics, University of Zanjan, University Blvd., 45371-38791 Zanjan, Iran; 2 Department of Physics, Institute for Advanced Studies in Basic Sciences (IASBS), 45137-66731 Zanjan, Iran; 3 Optics Research Center, Institute for Advanced Studies in Basic Sciences (IASBS), 45137-66731 Zanjan, Iran

**Keywords:** Nonlinear optics, Quantum optics

## Abstract

We investigate the reforming of a plane wave into a periodic waveform in its propagation through a structural asymmetry four-level quantum dot molecule (QDM) system that is induced by an inter-dot tunneling process and present the resulting tunneling-induced Talbot effect. The tunneling process between two neighborhood dots is provided with the aid of a gate voltage. Using a periodic coupling field the response of the medium to the propagating plane probe beam becomes periodic. The needed periodic coupling field is generated with the interference of two coherent plane waves having a small angle and propagating almost parallel to the probe beam direction. In the presence of the tunneling effect of an electron between two adjacent QDs, for the probe beam propagating through the QDM system, the medium becomes transparent where the coupling fields interfere constructively. As a result, the spatial periodicity of the coupling field modulates the passing plane probe beam. We determine the minimum length of the QDM system to generate a periodic intensity profile with a visibility value equal to 1 for the probe field at the exit plane of the medium. It is also shown that by increasing the propagation length of the probe beam through the QDM medium, the profile of the maximum intensity areas becomes sharper. This feature is quantified by considering a sharpness factor for the intensity profile of the probe beam at the transverse plane. Finally, we investigate free space propagation of the induced periodic field and present the Talbot images of the tunneling-induced periodic patterns at different propagation distances for different values of the QDM medium lengths. The presented dynamically designing method of the periodic coherent intensity patterns might find applications in science and technology. For instance, in optical lithography, the need to use micro/nanofabricated physical transmission diffraction gratings, in which preparation of them is expensive and time-consuming, can be eliminated.

## Introduction

The Talbot effect so-called self-imaging phenomenon appears in the diffraction of a spatially coherent wave from a periodic aperture such as a grating^1,2^. This effect occurs in the near-field diffraction regime, in which the same and rescaled copies of the light distribution immediately after the aperture reproduce at certain distances from the aperture. In this effect, the exact images of the aperture are known as Talbot-images, and the distance between two successive Talbot-images is called Talbot distance. At the half-way between two successive Talbot-images, the light distribution is similar to the light distribution immediately after the aperture with a lateral shift of half of the period. These images are called half-Talbot images. Furthermore, complicated sub-images having higher spatial frequencies form between every two adjacent Talbot and half-Talbot images which are known as fractional-Talbot images. Due to the cheap equipment needed and ease of the use of the Talbot effect, it has gained many applications in optics and interdisciplinary areas, such as in optical testing and metrology^3^, Talbot array illumination^4^, atmospheric turbulence studies^5^, singular optics including characterization^6^ and multiplication^7^ of vortex beams. Although the Talbot effect was initially observed in optics, in recent decades, it attracted the interest of many researchers in other areas of physics, and similar processes have also been reported with other physical waves, such as non-classical light^8^ and atomic waves^9–11^, X-ray phase imaging^12^, Bose-Einstein condensates^13^, metamaterials^14^, exciton polaritons^15^, surface plasmonic^16^, coupled lasers^17^ and waveguide arrays^18^ systems. The Talbot effect was also reported for mechanical waves such as water waves^19^ and acoustic waves^20^. In addition, the self-imaging phenomenon has been observed for other periodic features in physics including temporal Talbot effect^21^, quantum Talbot effect^22^, nonlinear Talbot effect^23^, angular Talbot effect^24^, and PT-symmetric Talbot effect^25^. Another aspect of the Talbot effect is the Talbot carpet. For a conventional periodic structure, the intensity pattern in the longitudinal plane known as Talbot carpet demonstrates the formation of self-images and sub-images in different propagation distances^26^. It is shown that for a radial grating, the Talbot carpet forms at the transverse planes^27^.

One of the top issues in the Talbot phenomenon is the design of periodic structures so that high-resolution images can be produced at the fractional-Talbot planes. For example, high-contrast sub-images of a binary grating can be generated at quarter-Talbot distances only by choosing suitable opening numbers for the grating^28^. This feature can be used in various domains such as lithography. Here we present a parametric method to produce a controllable periodic intensity pattern at the exit plane of a QDM system. We also investigate the resulting Talbot effect and it is shown that the intensity profile at the exit plane can be controlled by the medium parameters such as the tunneling parameter. The nonlinear optical properties of quantum systems can be controlled by the quantum coherence and quantum interference^29^. One of the most important phenomena due to quantum interference is electromagnetically induced transparency (EIT)^30,31^. The EIT phenomenon has many potential applications such as slow light^32,33^, light storage^34^, electromagnetically induced focusing (EIF)^35^, and electromagnetically induced grating (EIG)^36^. The latest case is the basis of our study. In an EIT-based quantum system, the optical response of the medium can be periodic when the traveling coupling field is replaced by a standing one. So, the probe field passing through such a system alternatively experiences the absorption and transparency of the medium.

In recent years, the Talbot effect has been studied in the EIG-based atomic systems. As the first experimental study, Zhang et al. showed the Talbot effect in the nonlinear photonic crystals, due to the periodic structure of the crystals^23^. Then, the electromagnetically induced Talbot effect (EITE) has been theoretically studied in a nonmaterial grating^37^. It was shown that the Talbot effect can be seen for a probe field passed through a three-level $\Lambda$-type atomic system, which is interacted with a standing coupling field. Recently, the Talbot effect was investigated based on the electromagnetically induced lattice in four-level *Y*-configuration and three-level cascade-type ultra-cold atoms^38,39^. More recently, the EITE was experimentally and theoretically obtained in a four-level ladder-type atomic system^40^. The Talbot carpet in the suggested atomic model perfectly defined both integer and fractional EITE. It seems that a simple controlling parameter for the EITE is needed. Quantum dot molecule (QDM) systems which have been widely studied in the field of quantum optics^41–45^, can be used to realize EITE.

In this work, we propose a novel mechanism to study the EITE. An ensemble of the structural asymmetry QDMs is introduced which has a periodic behavior for the input probe field. This periodicity is created by a strong standing coupling field applied to the QDM system. In our model, an external gate voltage can provide the possibility of tunneling for the conduction electrons between two quantum dots (QDs). It is shown that the inter-dot tunneling process induces the Talbot effect for the output probe field. The contrast of the self-images and fringes sharpness can be controlled by the value of tunneling parameter, the strength of coupling field and medium length of the QDM system, in which for certain values of these parameters of the QDM system, high contrast and highly sharped intensity fringes/spots can be generated over the self-images. Moreover, the Talbot carpet is calculated and shown that the images and sub-images of the output probe field appear along the propagation direction. Our results can be used for broad applications in the imaging of quantum systems. To the best of our knowledge, this is the first work that introduces a rather simple dynamical controlling parameter for control of the EITE using QDMs. It is demonstrated that the visibility and fringe sharpness of the output probe field, as well as the Talbot carpet patterns, can be simply manipulated by changing the gate voltage.

## Theoretical framework

### The QDM system

We introduce a uniform ensemble of the structural asymmetry QDMs in which each of the molecules consists of closely spaced coupling of two quantum dots. So, an electron can pass through the potential barrier between quantum dots via the inter-dot tunneling. As a realistic example, we consider a lateral quantum coupling between two self-assembled (In,Ga)As/GaAs quantum dots with different band structures. It can be produced by a unique combination of molecular beam epitaxy and atomic layer precise in situ etching on GaAs(001) substrates which can provide a low density of about $5\times 10^7$ cm^−2^ homogeneous ensemble of QDMs consisting of two dots aligned along the $[1\bar{1}0]$ direction^46^. However, the combination of the lateral and vertical growing techniques should be used to generate a three-dimensional quantum dot configuration^47^. The typical lateral size of each QD is about 35 nm. The inter-dot barrier thickness should be considered few nanometers (less than 8 nm) to allow significant inter-dot electron tunneling to occur. The inter-dot coupling can be controlled by applying an external electric field along with the molecular (coupling) axis via simple Schottky contacts. For further experimental details of the fabrication technique, one can refer to^48^.

**Figure 1:**
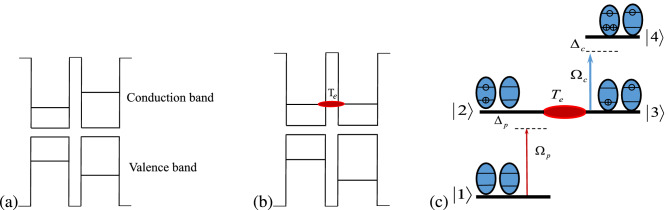
The energy levels of the QDM system before (**a**) and after (**b**) applying the external voltage. (**c**) The energy diagram of the four-level ladder type QDM system and two applied laser fields. The electron and hole are shown by $\ominus$ and $\oplus$, respectively.

**Figure 2:**
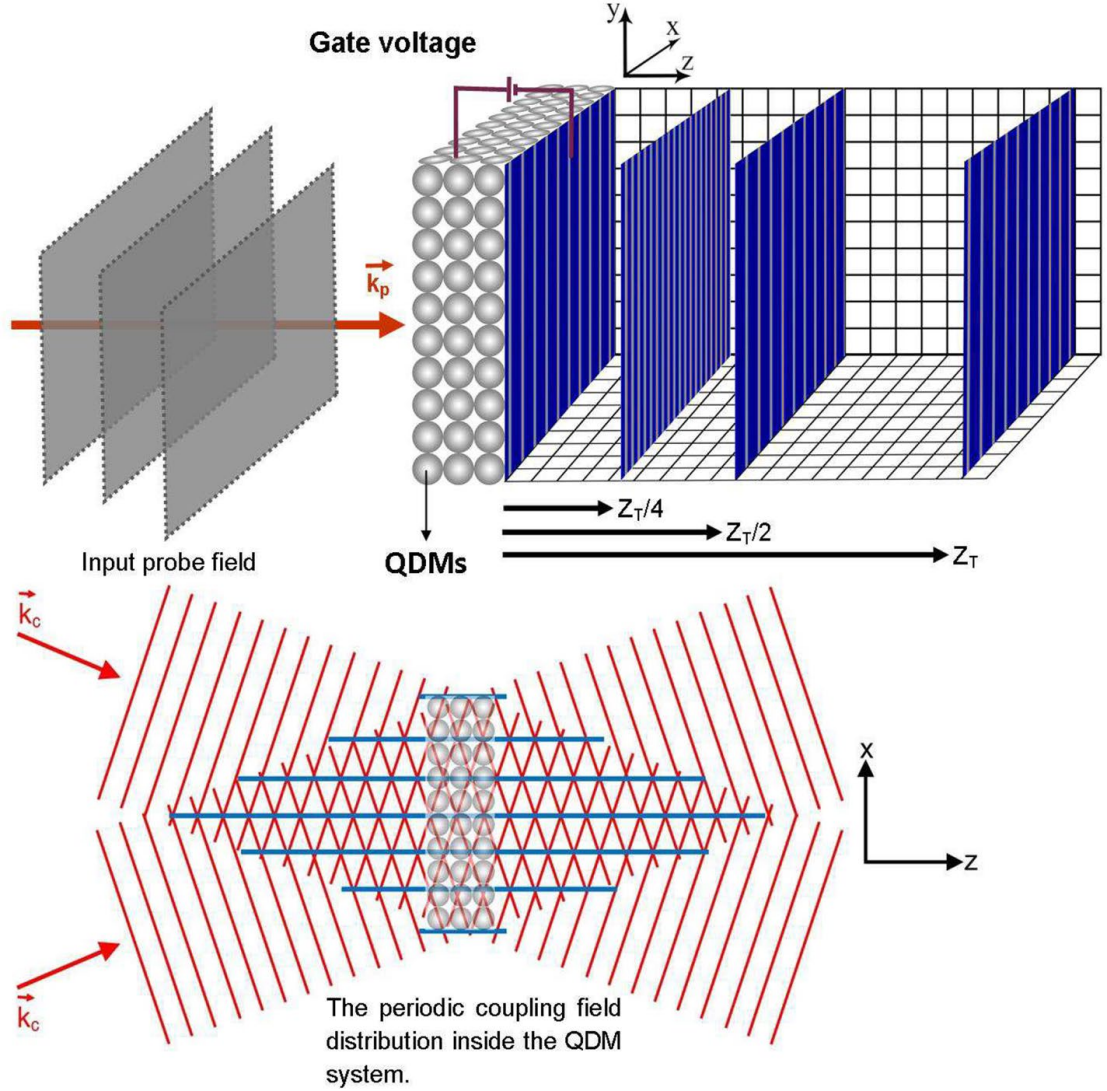
A possible arrangement of the QDM system including the input probe field with the plane wavefront, standing coupling field, the output probe field profile and its image patterns.

The band structure of the asymmetric QDM before (a) and after (b) applying the external voltage is shown in Fig. 1. The upper level of the left QD’s conduction band has a different energy from the upper level of the right QD’s conduction band. The energy difference between the upper levels can be removed by applying an external voltage to the QDM. Therefore, the electron tunneling effect occurs between the near-degenerate states, as shown in Fig. 1b. Four energy levels of the QDM is shown in Fig. 1c. The Dirac bra-ket notation $|e_L,h_L\rangle |e_R,h_R\rangle$ is used to show the excitonic states of the QDM. In the asymmetric QDMs, the central frequencies of the transition in left and right quantum dots are different. So, the selective excitation can be controlled by the frequency of the coupling fields. The ground state, $|1\rangle =|0,0\rangle |0,0\rangle$, defines a level in which two QDs are in valance band. The state $|2\rangle =|1,1\rangle |0,0\rangle$ stands for the level in which an electron is excited to the conduction band in left QDs to generate an exciton. The state $|3\rangle =|0,1\rangle |1,0\rangle$ illustrates the transfer of electron via inter-dot tunneling to the conduction band of the second QD and generation of an indirect exciton. Finally, the state $|4\rangle =|1,2\rangle |1,0\rangle$ describes the biexciton which is established by exciting the electron to the conduction band of first QD^49,50^. The spontaneous emission rates from the $\vert 2\rangle$ and $\vert 4\rangle$ levels are indicated by $\Gamma _{2}$ and $\Gamma _{4}$, respectively. A weak probe field with Rabi frequency $\Omega _{p}$ and frequency $\omega _{p}$ is applied to the $\vert 1\rangle \leftrightarrow \vert 2\rangle$ transition. The frequency difference between the probe field and the excited transition is indicated by $\Delta _{p}=\omega _{p}-\omega _{21}$. The transition $\vert 3\rangle \leftrightarrow \vert 4\rangle$ is driven by a standing coupling field, which is generated by the interference of two similar plane fields propagating in nearly opposite directions. It should be pointed out that the spatial period of the interference pattern inside the QDM medium depends on the angle between the wave vectors of the interfering fields. The Rabi frequency of the coupling field is denoted by $\Omega _{c}$ and $\Delta _{c}=\omega _{c}-\omega _{43}$ describes the frequency detuning of the coupling field and related transition. It should be noted that the central frequency of transition $|i\rangle \leftrightarrow |j\rangle$ is defined by $\omega _{ij}=\omega _i-\omega _j$ where $\omega _i=E_i/\hslash$ indicates the frequency corresponding to the $|i\rangle$ state with the energy value of $E_i$. The Rabi frequency is defined as $\Omega =\mathbf {\mu }\cdot \mathbf {E}/\hslash$, in which $\mathbf {\mu }$ and $\mathbf {E}$ are the induced dipole moment of the corresponding transition and the electric field’s amplitude, respectively, and $\hslash$ is the Planck’s constant. The induced dipole moment in QDMs takes the value of about $1.6 \times 10^{-19}$C nm^51^. So, the electric field is in the order of $10^{3}$ V/m considering $\Omega \sim$ μeV. Note that the coupling field is a standing wave that its Rabi frequency can be written as $\Omega _{c}=\Omega _{c0}\cos (\pi x/d)$. Here, the spatial period of the standing wave is denoted by *d* which depends on the angle between the two coupling fields. A possible arrangement of the QDM system interacting with the applied fields is presented in Fig. 2. The input probe field with the plane wavefront, $\mathbf {k}_p$, enters the QDM medium which has become periodic via the standing coupling field, $\mathbf {k}_c$. The output probe field profile and its image patterns are shown at different distances from the QDM medium. For a better understanding of the suggested model, the QDM medium is exaggeratedly drawn large in which each sphere shows a QDM with coupled energy states. Here, an external gate voltage can supply the electron tunneling process between the two QDs of the QDM.

The interaction Hamiltonian of the QDM system interacting with the applied fields, in the electric-dipole and rotating-wave approximations, leads to

1 $$\begin{aligned} H=-\hslash [\Omega _{p}e^{-i\Delta _{p}t}|1\rangle \langle 2|+\Omega _{c}e^{-i\Delta _{c}t}|3\rangle \langle 4|]+T_{e} e^{i\omega _{23}t}|2\rangle \langle 3|+C.C., \end{aligned}$$

which is written in interaction picture^52^.

The matrix form of the Hamiltonian is given by

2 $$\begin{aligned} H=\left( \begin{array}{c c c c} 0 &{} -\Omega _{p}e^{-i\Delta _{p}t} &{} 0 &{} 0 \\ -\Omega _{p}^{*}e^{i\Delta _{p}t} &{} 0 &{} T_{e} e^{i\omega _{23}t} &{} 0 \\ 0 &{} T_{e} e^{-i\omega _{32}t} &{} 0 &{} -\Omega _{c}e^{-i\Delta _{c}t} \\ 0 &{} 0 &{} -\Omega _{c}^{*}e^{i\Delta _{c}t} &{} 0 \\ \end{array} \right) , \end{aligned}$$

where $T_{e}$ is the tunneling parameter in the QDM system.

To study the time evolution of the QDM system, we obtain the Bloch equations of the density matrix operator, using the von Neumann equation^53^,

3 $$\begin{aligned} \dot{\rho }_{11}= & {} i(\rho _{21}\Omega _{p}-\rho _{12}\Omega _{p}^{*})+\Gamma _{2}\rho _{22},\nonumber \\ \dot{\rho }_{12}= & {} i((\rho _{22}-\rho _{11})\Omega _{p}+\Delta _{p}\rho _{12}-\rho _{13}T_{e})-\frac{\Gamma _{2}}{2}\rho _{12},\nonumber \\ \dot{\rho }_{13}= & {} i((\Delta _{p}-\omega _{23})\rho _{13}-\rho _{12}T_{e}+\rho _{23}\Omega _{p}-\rho _{14}\Omega _{c}^{*}),\nonumber \\ \dot{\rho }_{14}= & {} i((\Delta _{c}+\Delta _{p}-\omega _{23})\rho _{14}-\rho _{13}\Omega _{c}+\rho _{24}\Omega _{p})-\frac{\Gamma _{4}}{2}\rho _{14},\nonumber \\ \dot{\rho }_{22}= & {} i(\rho _{12}\Omega _{p}^{*}+(\rho _{32}-\rho _{23})T_{e}-\rho _{21}\Omega _{p})-\Gamma _{2}\rho _{22},\nonumber \\ \dot{\rho }_{23}= & {} i((\rho _{33}-\rho _{22})T_{e}-\omega _{23}\rho _{23}+\rho _{13}\Omega _{p}^{*}-\rho _{24}\Omega _{c}^{*})-\frac{\Gamma _{2}}{2}\rho _{23},\nonumber \\ \dot{\rho }_{24}= & {} i((\Delta _{c}-\omega _{23})\rho _{24}+\rho _{14}\Omega _{p}^{*}+\rho _{34}T_{e}+\rho _{23}\Omega _{c})-\frac{(\Gamma _{2}+\Gamma _{4})}{2}\rho _{24},\nonumber \\ \dot{\rho }_{33}= & {} i((\rho _{23}-\rho _{32})T_{e}+\rho _{43}\Omega _{c}-\rho _{34}\Omega _{c}^{*})+\Gamma _{4}\rho _{44},\nonumber \\ \dot{\rho }_{34}= & {} i((\rho _{44}-\rho _{33})\Omega _{c}-\Delta _{c}\rho _{34}+\rho _{24}T_{e})-\frac{\Gamma _{4}}{2}\rho _{34},\nonumber \\ \dot{\rho }_{44}= & {} -(\dot{\rho }_{11}+\dot{\rho }_{22}+\dot{\rho }_{33}). \end{aligned}$$

The steady state susceptibility of the medium, $\chi$, corresponding to the coherence term, $\rho _{21}$, for $\Delta _c=\omega _{23}=0$ is obtained as

4 $$\begin{aligned} \chi (x)=\frac{N |\mu |^{2}}{2\hslash \varepsilon _{0}\Omega _{p}}\rho _{21}=\frac{N |\mu |^{2}}{2\hslash \varepsilon _{0}}\frac{(\Gamma _{4}+i\Delta _{p})\Delta _{p}-i\Omega _{c0}^{2}\cos ^{2}(\pi x/d)}{D}, \end{aligned}$$

where *N* indicates the density number of QDMs and

$$\begin{aligned} D=-(\Gamma _{2}+i\Delta _{p})\Omega _{c0}^{2}\cos ^{2}(\pi x/d)+(\Gamma _{4}+i\Delta _p)(-T_e^2+\Delta _p(-i\Gamma _{2}+\Delta _p)). \end{aligned}$$

It can be observed that Eq. (4) reduces to

5 $$\begin{aligned} \chi (x)=\frac{N |\mu |^{2}}{2\hslash \varepsilon _{0}}\frac{i\Omega _{c0}^{2}\cos ^{2}(\pi x/d)}{\Gamma _{4}T_e^2+\Gamma _{2}\Omega _{c0}^{2}\cos ^{2}(\pi x/d)}, \end{aligned}$$

considering $$\Delta _{p}=0$$.

It is well known that the real and imaginary parts of the susceptibility represent the dispersion and absorption of the medium to the applied field. Since the coupling field is a standing wave with periodic Rabi frequency, the QDM medium shows a periodic response to the input probe field. So, the probe field experiences a periodic absorption during passing through the medium, leads to the periodic output probe field. However, for the zero tunneling parameter, the susceptibility does not depend on the coupling field Rabi frequency. Then, the output probe field profile becomes uniform which cannot have the Talbot images in the space. To calculate the output probe field, we use the Maxwell equation of a weak field, in the slowly varying envelope approximation, which has the form

6 $$\begin{aligned} \frac{\partial E_{p}}{\partial z}=ik_{p} \chi E_{p}. \end{aligned}$$

Here, the weak probe field is considered to be a plane-wave and $k_{p}$ indicates the probe field’s wave vector value. Solving Eq. (6) leads to the output probe field as

7 $$\begin{aligned} E_{p}(x,z=L)=E_{p}(z=0)exp\{[-k_{p}Im(\chi )+ik_{p}Re(\chi )]L/2\}, \end{aligned}$$

where *L* and $E_{p}(z=0)$ are the length of the QDM medium and the constant amplitude of the input probe field, respectively. According to Eqs. (4) and (7), the intensity of the output probe field has a periodic structure along the *x*-direction for the case of $\Delta _p=0$ as below

8 $$\begin{aligned} I_{p}(z=L)=|E_{p}(z=L)|^2=|E_{p}(z=0)|^2exp(\frac{-k_{p}\Omega _{c0}^{2}\cos ^{2}(\pi x/d)L}{(\Gamma _{4}T_e^2+\Gamma _{2}\Omega _{c0}^{2}\cos ^{2}(\pi x/d))}), \end{aligned}$$

which is completely tunneling-dependent. It is clear that the periodicity of the output probe field is destroyed in the absence of the tunneling effect.

To characterize the brightness contrast of the intensity pattern of the induced periodic waveform at the end of the QDM system, we introduce the visibility parameter

9 $$\begin{aligned} \textit{V}=\frac{I_{max}-I_{min}}{I_{max}+I_{min}}=\frac{1-exp(-k_{p}L\Omega _{c0}^{2}/(\Gamma _{4}T_e^2+\Gamma _{2}\Omega _{c0}^{2}))}{1+exp(-k_{p}L\Omega _{c0}^{2}/(\Gamma _{4}T_e^2+\Gamma _{2}\Omega _{c0}^{2}))}, \end{aligned}$$

where $I_{max}$ and $I_{min}$ are the magnitudes of the light intensity at the neighboring maximum and minimum of the intensity pattern. It can be deduced from Eq. (9) that the visibility of the output probe field pattern is decreased for greater values of the tunneling parameter and vice versa. This behavior is physically rooted in the linewidth of the EIT window in the optical spectrum, which becomes narrow in the presence of a weak tunneling effect^42^.

The sharpness of the induced periodic waveform at the end of the QDM system can be characterized by the ratio of separation between adjacent intensity maxima (*d*) to the width of an individual intensity maximum (*w*) as

10 $$\begin{aligned} \textit{F}=d/w=\frac{\pi }{2}[\sin^{-1}\left( \frac{T_e}{\Omega _{c0}}\sqrt{\frac{\Gamma _{4}}{k_{p}L-\Gamma _{2}}}\right) ]^{-1}. \end{aligned}$$

We call the value of $\textit{F}$ “finesse of fringes” of the produced periodic intensity pattern. It should be mentioned that increasing the value of the tunneling parameter leads to a reduction for the finesse of fringes, which is related to the tunneling-dependent behavior of the absorption spectrum for the suggested QDM system.

Using Eq. (10), the finesse of fringes (fringe sharpness) of the induced periodic waveform at the end of the QDM system is characterized (Fig. 7). The same sharpness can be observed over the diffracted pattern at the self-image planes. The conventional way to generate the spot grid is using a microlens array, whose numerical aperture is limited by the manufacturing capability. Therefore, it is important to determine how the sharpness of the fringes/spots (for a 2D case) produced in a QDM system decreases under propagation and what is the minimum achievable fringe/spot width?

For a microlens having a diameter of entrance pupil *D* and focal length *f* under a plane wave illumination the radius of intensity spot at the focal plane is given by $w=1.22\lambda f/D$, where $\lambda$ is the wavelength of the illuminating beam. The *f*-number of a microlens, defined by $N=f/D$ is estimated to be around 1.4 and the numerical aperture of a microlens in a normal use in air ($n=1$) is given by $NA=\sin(\theta )= \sin[arc\tan (D/2f)]$. For typical values of *D* = 4 mm, *f* = 6 mm and $\lambda$ = 500 nm, we have an intensity spot at the focal plane with a radius of 400 nm and the system numerical aperture is $NA=\sin[arc\tan (0.33)]=0.314$.

Now, let us estimate the minimum achievable fringe/spot width in a QDM system and determine the corresponding numerical aperture by calculating the divergence angle of the intensity spot under propagation at the vicinity of the Talbot planes. We calculate full width at half maximum (*FWHM*) of the fringe intensities at the vicinity of the Talbot plane. For typical values of system parameter $L=2.5 \,\upmu$m, $\Omega _{c0}=0.5\Gamma$, $T_e=0.5\Gamma$, $\lambda =870\, nm$, and $d=10\, \upmu$m, we have $FWHM=1.5\, \upmu$m at the Talbot plane. Our calculation shows that in a length $Z=1.05Z_T$ the width of intensity spots increases 2.7 times ($FWHM: 1.5\, \upmu {\text{m}}\rightarrow 4 \,\upmu$m). This is equal to a numerical aperture of 0.978 for the system, which is more than the value of *NA* for a typical microlens. Such convergence of the beam at the vicinity of the light spot (at the self-image plane) provides a suitable beam for a stronger multi-particle optical tweezer with high restoring force.

In the following, we study the Talbot effect for the output weak probe field in our suggested model. Note that the length of the medium is chosen so small to prevent the self-imaging effect inside the medium.

### Talbot effect

The output surface of the QDM medium can be considered as a diffraction aperture. The Fresnel diffraction integral, in the paraxial approximation, for the output probe field with wavelength $\lambda _{p}$, is given by

11 $$\begin{aligned} \psi _{p}(X,Z)=\frac{e^{ik_{p}Z}}{\sqrt{\lambda _{p} Z}}\int _{-\infty }^{+\infty }E_{p}(x,L)\exp [ik_{p}(x-X)^{2}/2Z]dx, \end{aligned}$$

where *Z*, *x*, and *X* are the place of the observation plane from the output surface, the aperture and observation planes’ coordinates, respectively. Since the output probe field has an oscillating structure and can be written as the Fourier series, Eq. (11) can be recast into

12 $$\begin{aligned} \psi _{p}(X,Z)\propto \sum _{-\infty }^{\infty }C_{n}\exp \left( \frac{-i\pi \lambda _{p}n^2Z}{d^2}\right) \exp \left( \frac{i2n\pi X}{d}\right) , \end{aligned}$$

which describes the traditional Talbot effect. The details of the simple derivation can be found in Ref.^37^. Here, $C_{n}$ is the Fourier coefficient. The output probe field’s self-imaging planes, so-called Talbot planes, are defined by $Z_{T}=2m d^{2}/\lambda _{p}$, where *m* is the positive integer number of the Talbot planes. The self-images of the output probe field at the fractional Talbot planes are shifted by half a period with respect to the Talbot planes. However, the number of probe field oscillations increase at the quarter Talbot planes. So the Talbot effect is induced in our suggested system. It is worth noting that the Talbot effect is just obtained in the presence of the tunneling effect.

Now, it is worth considering some specific features of the quarter-Talbot images. We specify the diffraction patterns observing at the quarter-Talbot distances, which are halfway between the Talbot planes and their nearest half-Talbot planes, namely, $Z_{QT;n} =(2n - 1) \frac{Z_T}{4}$, $n =1; 2; 3;\ldots$. By replacing $Z = Z_{QT;n}$ in equation (12), and after a bit calculation (see a similar calculation of Section 3A of Ref.^28^), the light beam complex amplitude can be divided into two real and imaginary parts with the fundamental periods of *d*/2 and *d*, respectively. As a result, the fundamental periods of the intensity and phase patterns at the quarter-Talbot distances are equal to *d*/2 and *d*, respectively. Therefore, the period of the complex amplitude is still *d*. The quarter-Talbot distances are a very special case of fractional Talbot distances. At fractional locations $Z_F = \frac{k}{l}Z_T$, where *k* and *l* are coprime integers, the complex amplitude of the propagated light can be presented as equations (29) and (30) of the same reference^28^.

## Results and discussions

**Figure 3:**
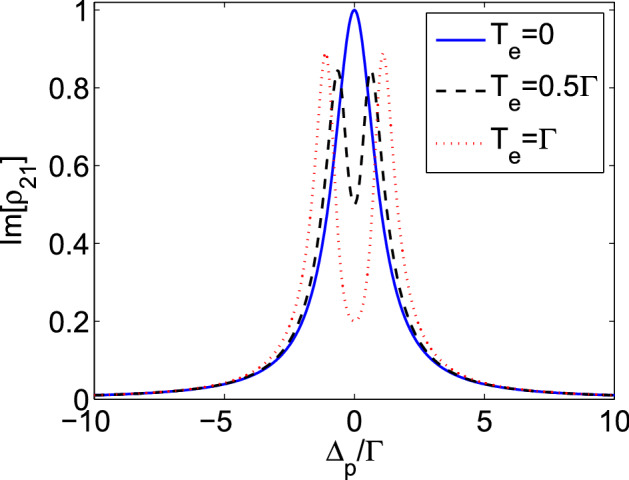
Imaginary part of $\rho _{21}$ as a function of $\Delta _{p}/\Gamma$. Used parameters are $\Gamma _{2}=\Gamma _{4}=\Gamma$, $\Omega _{p}=0.1\Gamma$, $\Omega _{c}=0.5\Gamma$, and $\Delta _{c}=0$.

Now, we are going to study the effect of the tunneling process on the self-imaging of an oscillating electric field in the QDM system by using Eqs. (4), (7), and (11). At first, we investigate the absorption spectrum of the QDM system for different tunneling parameter values. Figure 3 shows the imaginary part of $\rho _{21}$ as a function of $\Delta _{p}$ for $T_{e}=0$ (solid), $0.5\Gamma$ (dashed) and $\Gamma$ (dotted). Used parameters are $\Gamma _{2}=\Gamma _{4}=\Gamma$, $\Omega _{p}=0.1\Gamma$, $\Omega _{c}=0.5\Gamma$, and $\Delta _{c}=0$. Note that all frequency parameters including $\Omega _{p}$, $\Omega _{c}$, $\Delta _{p}$, and $T_e$ are scaled by $\Gamma$ which is in the order of $1\, \upmu$eV for the (In,Ga)As/GaAs QDs^54^. In the absence of the tunneling effect, the excited electron of the left QD has no way to transfer to the right QD. So, an absorption peak appears at the resonant frequency. However, increasing the tunneling parameter from zero up to its maximum value makes the QDM medium more transparent. In some cases, for the maximum value of the tunneling parameter, complete transparency can be obtained, which is called tunneling-induced transparency (TIT)^55^.

**Figure 4:**
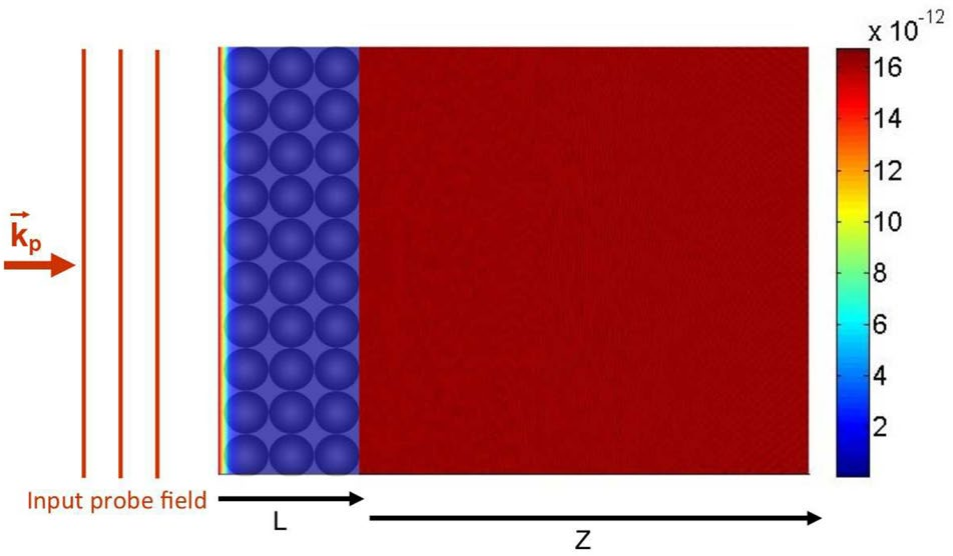
The propagation of the probe field amplitude inside the QDM medium, $|E_p(x,z)/E_p(0)|$, and outside it, $|\psi _p(X,Z)/E_p(0)|$, in the absence of the tunneling effect, $T_{e}=0$. Used parameters are $\Omega _{c0}=0.5\Gamma$, $d=10\,\upmu m$, $\lambda _p=870$ nm, and $L=6.9\, \upmu$m under the multi-photon resonance condition, $\Delta _p=\Delta _c=0$.

**Figure 5:**
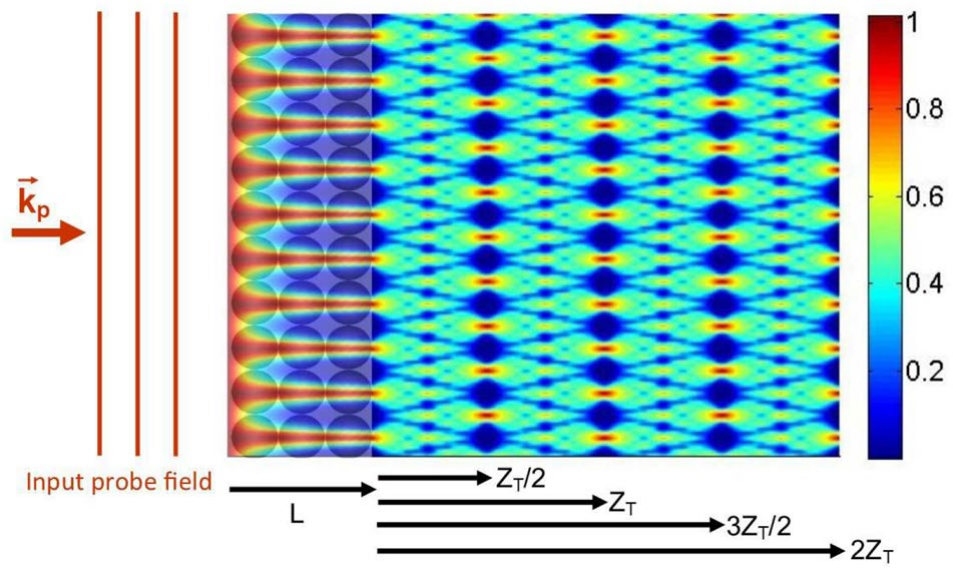
The propagation of the probe field amplitude inside the QDM medium, $|E_p(x,z)/E_p(0)|$, and outside it, $|\psi _p(X,Z)/E_p(0)|$, for the maximum value of the tunneling parameter, $T_{e}=\Gamma$. Other used parameters are the same as in Fig. 4.

**Figure 6:**
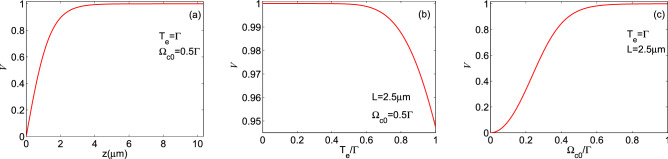
The visibility of the induced periodic waveform as a function of (**a**) medium length (for $T_e=\Gamma$ and $\Omega _{c0}=0.5\Gamma$), (**b**) tunneling parameter (for $L=2.5\, \upmu$m and $\Omega _{c0}=0.5\Gamma$), and (**c**) strength of the coupling field (for $T_e=\Gamma$ and $L=2.5\, \upmu$m).

A general study on the Talbot effect is possible at different distances from the output surface of the medium which leads to the Talbot carpet. Figure 4 illustrates the propagation of the probe field amplitude inside the QDM medium, $|E_p(x,z)/E_p(0)|$, and outside it, $|\psi _p(X,Z)/E_p(0)|$, in the absence of the tunneling effect, $T_e=0$. It should be noted that, in our calculations, the integration limits of the integral in Eq. (11) are considered from $-20d$ up to 20*d*. Used parameters are $\Omega _{c0}=0.5\Gamma$, $d=10\,\upmu$m, $\lambda _p=870$nm, and $L=6.9\, \upmu$m under the multi-photon resonance condition, $\Delta _p=\Delta _c=0$. As it is also expected from Eq. (5) and Fig. 3, the necessary condition for the generation of the Talbot effect is the tunneling induced transparency, which is switched to the spatially independent absorption peak in the absence of the tunneling effect. So, the probe field experiences high absorption passing through the QDM medium. It is clear that the weak probe field keeps its planar form out of this medium. In Fig. 5, the propagation of the probe field amplitude is investigated inside the QDM medium and outside it, for the maximum value of the tunneling parameter, $T_{e}=\Gamma$. Other used parameters are the same as in Fig. 4. It can easily be seen that the uniform probe field profile changes to a periodic pattern so that the Talbot effect occurs for the output probe field. It is shown that the self-imaging of the output probe field is created at the Talbot planes. The output probe field’s self-images at $Z=Z_{T}/2$ and $Z=3Z_{T}/2$ are shifted half a period with respect to the output probe field. However, the self-images at $Z=Z_{T}$ and $Z=2Z_{T}$ are the same as the output probe field. It is demonstrated that the periodic pattern of the output probe field’s Fresnel diffraction has a higher frequency and different oscillation properties at the quarter Talbot planes. Moreover, Fig. 2 symbolically shows the QDM medium under the applied laser fields which makes the output probe field profile as a periodic structure. The output probe field at the end plane of the QDM medium and some of its images along the propagation direction are plotted in two dimensions for the maximum value of the tunneling parameter. The first Talbot image, at $Z=Z_T$, and half Talbot image with a $\pi$ phase shift and the quarter Talbot images, at $Z=Z_T/2$ and $Z=Z_T/4$, are shown in Fig. 2. It is shown that the fractional Talbot image has a different periodicity with respect to the Talbot image whereas the first Talbot image is the same as the output probe field.

The visibility of the induced periodic waveform is plotted as a function of (**a**) medium length (for $T_e=\Gamma$ and $\Omega _{c0}=0.5\Gamma$), (**b**) tunneling parameter (for $L=2.5\, \upmu$m and $\Omega _{c0}=0.5\Gamma$), and (**c**) strength of the coupling field (for $T_e=\Gamma$ and $L=2.5\, \upmu$m), in Fig. 6. Other parameters are the same as in Fig. 4. Figure 6a,c show that the visibility reaches its maximum value by increasing the medium length and constant Rabi frequency of the coupling field. It is obvious that the interaction of two applied fields and the QDM medium has been raised when the length of the medium is chosen to be large enough. On the other hand, considering a stronger standing coupling field leads to a more complete absorption for the probe field at the anti-node positions of the coupling field. So, the intensity of the output periodic waveform becomes zero at these positions. Figure 6b demonstrates inducing the periodic structure for the probe field by the tunneling effect with the unit value of visibility. However, increasing the tunneling parameter decreases the visibility of the induced periodic waveform, as deduced from Eq. (9). Since the small values of the tunneling parameter lead to the exact zero value for the absorption at the nodes of the standing coupling field and the absorption peaks at its anti-nodes, the induced periodic waveform possesses the maximum value of visibility. However, increasing the value of the tunneling parameter results in larger linewidths for the tunneling-induced transparency and leads to decreasing the visibility of the output probe field.

**Figure 7:**
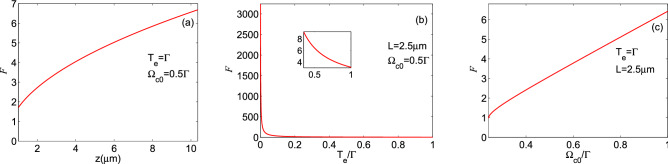
The finesse of fringes of the induced periodic waveform versus (**a**) medium length (for $T_e=\Gamma$ and $\Omega _{c0}=0.5\Gamma$), (**b**) tunneling parameter (for $L=2.5 \,\upmu$m and $\Omega _{c0}=0.5\Gamma$), and (**c**) strength of the coupling field (for $T_e=\Gamma$ and $L=2.5 \,\upmu$m).

In Fig. 7, we also illustrate the finesse of fringes of the induced periodic waveform versus (a) medium length (for $T_e=\Gamma$ and $\Omega _{c0}=0.5\Gamma$), (b) tunneling parameter (for $L=2.5\,\upmu$m and $\Omega _{c0}=0.5\Gamma$), and (c) strength of the coupling field (for $T_e=\Gamma$ and $L=2.5\,\upmu$m) under the same parameters of Fig. 4. It can easily be seen that the finesse of fringes goes up for the large amounts of medium length and constant Rabi frequency of the coupling field, while, it declines with increasing tunneling parameter. Our numerical results are consistent with the finesse of fringes characteristics expected from Eq. (10). It is interesting to note that the high visibility output probe field is accompanied by a large amount of finesse of fringes for the large length of the interaction, the small value of the tunneling parameter, and strong standing coupling fields. This noticeable finesse of fringes originates from the narrow EIT window and high absorption peaks in the optical spectrum as the coupling field has a periodic form.

**Figure 8:**
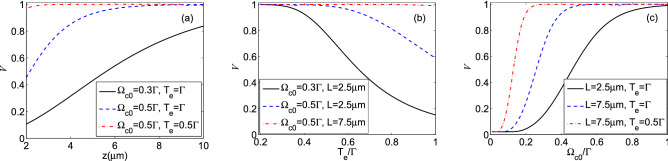
The visibility of the intensity profile of the quarter Talbot plane as a function of (**a**) medium length (for $\Omega _{c0}=0.3\Gamma$ and $T_e=\Gamma$ (solid curve), $\Omega _{c0}=0.5\Gamma$ and $T_e=\Gamma$ (dashed curve), and $\Omega _{c0}=0.5\Gamma$ and $T_e=0.5\Gamma$ (dash-dotted curve)), (**b**) tunneling parameter (for $\Omega _{c0}=0.3\Gamma$ and $L=2.5\, \upmu$m (solid curve), $\Omega _{c0}=0.5\Gamma$ and $L=2.5\, \upmu$m (dashed curve), and $\Omega _{c0}=0.5\Gamma$ and $L=7.5\, \upmu$m (dash-dotted curve)), and (**c**) strength of the coupling field (for $L=2.5\, \upmu$m and $T_e=\Gamma$ (solid curve), $L=7.5 \,\upmu$m and $T_e=\Gamma$ (dashed curve), and $L=7.5 \,\upmu$m and $T_e=0.5\Gamma$ (dash-dotted curve)). Other used parameters are the same as in Fig. 4.

Now, we explore how the QDM system’s characteristics can affect the visibility of the quarter Talbot image. Figure 8 presents the visibility of the intensity profile of the quarter Talbot plane as a function of (a) medium length (for $\Omega _{c0}=0.3\Gamma$ and $T_e=\Gamma$ (solid curve), $\Omega _{c0}=0.5\Gamma$ and $T_e=\Gamma$ (dashed curve), and $\Omega _{c0}=0.5\Gamma$ and $T_e=0.5\Gamma$ (dash-dotted curve)), (b) tunneling parameter (for $\Omega _{c0}=0.3\Gamma$ and $L=2.5 \,\upmu$m (solid curve), $\Omega _{c0}=0.5\Gamma$ and $L=2.5 \,\upmu$m (dashed curve), and $\Omega _{c0}=0.5\Gamma$ and $L=7.5\, \upmu$m (dash-dotted curve)), and (c) strength of the coupling field (for $L=2.5\, \upmu$m and $T_e=\Gamma$ (solid curve), $L=7.5\,\upmu$m and $T_e=\Gamma$ (dashed curve), and $L=7.5\, \upmu$m and $T_e=0.5\Gamma$ (dash-dotted curve)). Other used parameters are the same as in Fig. 4. In Fig. 8a, it can easily be seen that the small value of $\Omega _{c0}$ and strong tunneling effect are not good choices for achieving the maximum visibility of the quarter Talbot plane (solid curve). Increasing the strength of the standing coupling field (dashed curve) and decreasing the effect of the tunneling parameter (dash-dotted curve) can provide high visibility periodic pattern at the quarter Talbot plane. Such behavior for the visibility of the quarter Talbot plane’s pattern has been confirmed in Fig. 8b,c. It is shown that we can provide a high resolution periodic pattern in the quarter Talbot plane with a smaller period step whose visibility is completely controllable via the QDM medium length, tunneling parameter and coupling field’s strength. It should be addressed that the behavior of the quarter Talbot plane visibility follows a similar process corresponding to the output probe field. It is worth noting that adjusting the visibility of the quarter Talbot plane directly affects the visibility of the whole Talbot carpet.

**Figure 9:**
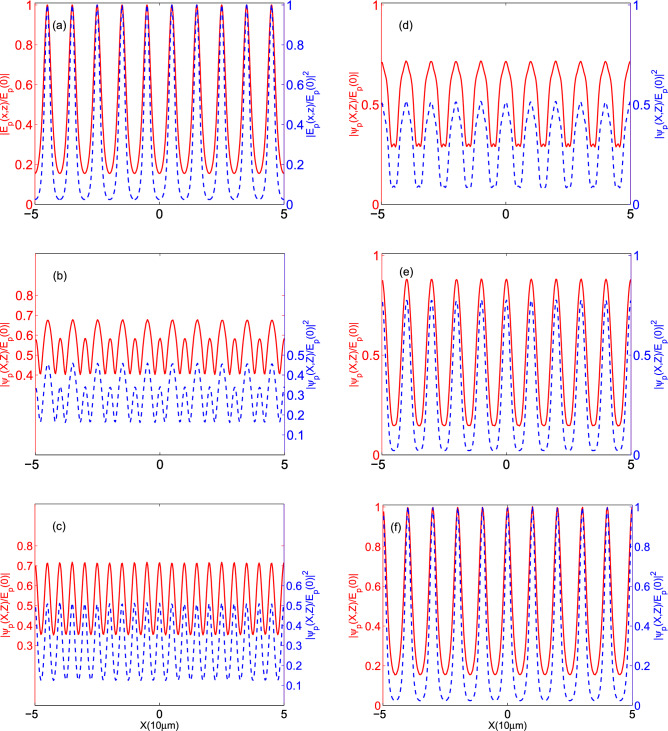
The profiles of (**a**) the output probe field at $z=L$ and its diffracted field at (**b**) $Z=Z_{T}/5$, (**c**) $Z=Z_{T}/4$, (**d**) $Z=3Z_{T}/8$, (**e**) $Z=9Z_{T}/20$, and (**f**) $Z=Z_{T}/2$. Solid (dashed) curves with the left (right) vertical axis demonstrate the amplitude (intensity) of the field in all panels. The selected set of parameters are $\Omega _{c0}=0.5\Gamma$, $L=2.5 \upmu$m, and $T_e=\Gamma$. The other used parameters are the same as in Fig. 4.

**Figure 10:**
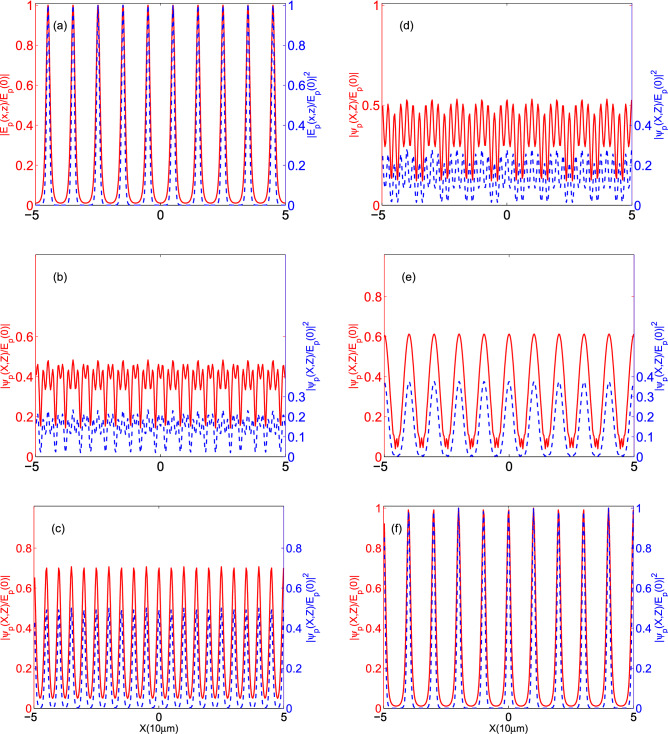
The profiles of (**a**) the output probe field at $z=L$ and its diffracted field at (**b**) $Z=Z_{T}/5$, (**c**) $Z=Z_{T}/4$, (**d**) $Z=3Z_{T}/8$, (**e**) $Z=9Z_{T}/20$, and (**f**) $Z=Z_{T}/2$ under the same parameters of Fig. 9 except for $T_e=0.5\Gamma$. Solid (dashed) curves with the left (right) vertical axis demonstrate the amplitude (intensity) of the field in all panels.

Here, we are going to investigate the behavior of the output probe field and its diffracted patterns at different distances from the end plane of the QDM medium under the specific sets of parameters. In Fig. 9, we plot the profiles of (a) the output probe field at $z=L$ and its diffracted field at (b) $Z=Z_{T}/5$, (c) $Z=Z_{T}/4$, (d) $Z=3Z_{T}/8$, (e) $Z=9Z_{T}/20$, and (f) $Z=Z_{T}/2$. Solid (dashed) curves with the left (right) vertical axis demonstrate the amplitude (intensity) of the field in all panels. The selected set of parameters are $\Omega _{c0}=0.5\Gamma$, $L=2.5\, \upmu$m, and $T_e=\Gamma$. The other used parameters are the same as in Fig. 4. It is founded out that the selected parameters lead to low visibility for the output probe field and also quarter Talbot image. However, a high visibility case can be generated by decreasing the tunneling parameter to $T_e=0.5\Gamma$. This result is shown in Fig. 10.

**Figure 11:**
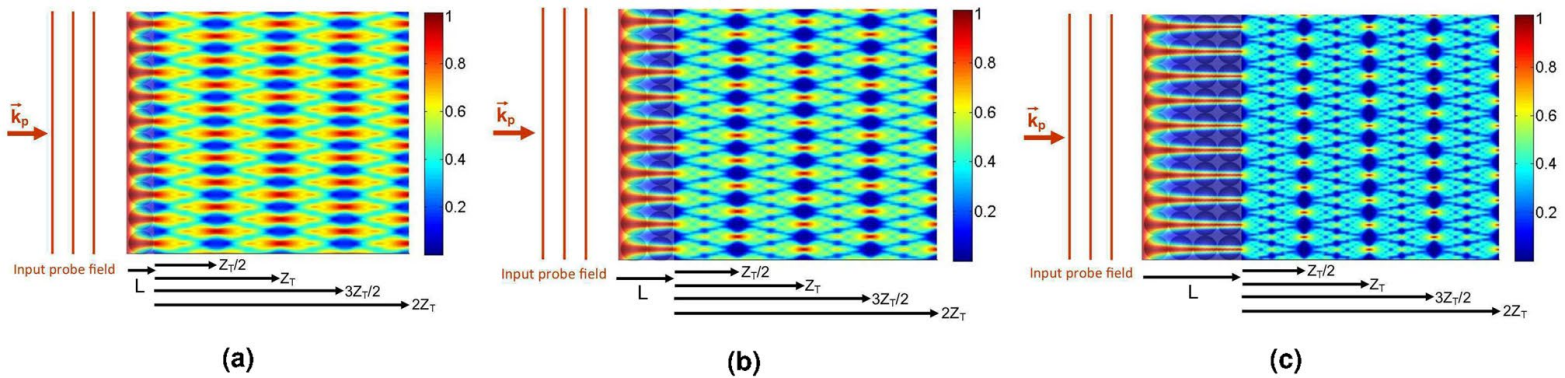
The propagation of the probe field amplitude inside the QDM medium, $|E_p(x,z)/E_p(0)|$, and outside it, $|\psi _p(X,Z)/E_p(0)|$, for different lengths of the QDM medium, i.e. (**a**) $L=2.5\, \upmu$m, (**b**) $L=5.25\, \upmu$m, and (**c**) $L=10.5\, \upmu$m. The tunneling parameter is fixed at $T_{e}=\Gamma$, whereas other parameter values are the same as in Fig. 4.

Finally, we obtain the Talbot carpet of the induced periodic waveform for better understanding the effect of the QDM medium characteristics on the visibility of the quarter Talbot image. Figure 11 presents the propagation of the probe field amplitude inside the QDM medium and outside it for different lengths of the QDM medium, i.e. (a) $L=2.5 \,\upmu$m, (b) $L=5.25\, \upmu$m, and (c) $L=10.5\, \upmu$m. The tunneling parameter is fixed at $T_{e}=\Gamma$, whereas other parameter values are the same as in Fig. 4. It can be seen that increasing the length of the QDM medium leads to the creation of a hight visibility doubled frequency image at the quarter Talbot plane which is in good agreement with the results of Fig. 8.

**Figure 12:**
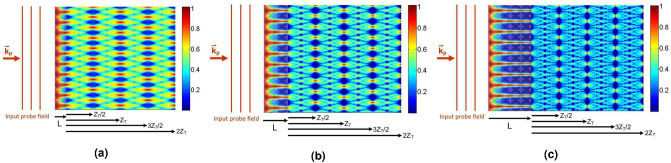
The propagation of the probe field amplitude inside the QDM medium, $|E_p(x,z)/E_p(0)|$, and outside it, $|\psi _p(X,Z)/E_p(0)|$, for $T_{e}=0.5\Gamma$ and different lengths of the QDM medium, i.e. (**a**) $L=1\, \upmu$m, (**b**) $L=2 \,\upmu$m, and (**c**) $L=4.25\, \upmu$m. Other used parameters are the same as in Fig. 4.

We also demonstrate the propagation of the probe field amplitude inside the QDM medium and outside it for $T_{e}=0.5\Gamma$ and different lengths of the QDM medium, i.e. (a) $L=1 \,\upmu$m, (b) $L=2 \,\upmu$m, and (c) $L=4.25 \,\upmu$m, in Fig. 12. Other used parameters are the same as in Fig. 4. It can be figured out that the value of the tunneling parameter has a noticeable impact on the quarter Talbot image visibility. In this manner, we can provide a suitable QDM medium to generate a doubled frequency electromagnetically induced grating with controllable visibility.

It should be mentioned that the spectral broadening due to the size distribution is the most important broadening mechanism in quantum dots. Production of an ensemble of QDs with the highest possible uniformity has a major role in this field. Several techniques have been introduced to reduce the inhomogeneous distribution of dot size^56^. A laser field experiences various detunings due to the non-uniformity of different QDs during interacting with them^43,57^. Then, the effect of size distribution can be understood via transmittance for different values of the detuning. As it was expected, the size distribution can affect the output probe field pattern generated by QDMs and reduces the visibility. Our results show that the visibility of the periodic intensity profile reduces by increasing the detuning of the applied fields. However, for spectral broadening less than the natural linewidth, the visibility does not noticeably change; So, it cannot destroy the main results of our study.

The presented adjustable method for the generation of periodic coherent intensity patterns has potential applications in the optical lithography. Here, we describe in brief but clearly the advantageous points of the QDMs-based method in comparison with the currently employed lithography methods. The Talbot lithography is a powerful lithographic technique presenting high-resolution and high-throughput micro/nanopatterning over large areas^58–61^. It utilizes extreme ultraviolet wavelengths, notably 13.5 nm. Improving the resolution and the throughput of the technique requires elaborate designs based on simulations and nanofabrication of transmission diffraction gratings on thin silicon nitride membranes. This process is time-consuming and expensive. On the other hand, since a physical grating has a constant period and a given transmission profile, its self-image at a given propagation distance has also a constant period and a given filling factor. In practice, these features seriously limit the use of self-images of physical gratings. For these reasons, in a wide range of potential applications this method has not found its true place and value. Therefore, we think that the QDMs-based method for the generation of periodic coherent intensity patterns with desired fill factor is a good candidate to get the place of physical gratings, the nanofabricated transmission diffraction gratings, in the optical lithography.

## Conclusion

We presented a simple method to generate an adjustable periodic intensity pattern from a plane wave under propagation through a QDM system. The period and intensity profile of the produced pattern can be adjusted with the variable physical parameters induced in the QDM system such as the value of gate voltage, length of the QDM system, and intensity and angle of the coupling waveforms producing the coupling field. It is shown that the medium becomes transparent for the probe beam propagating through the QDM system when an electron tunnels between two adjacent QDs. The tunneling effect dominantly occurs in the area the coupling field presents. As a result, the spatial periodicity of the coupling field modulates the propagating plane probe beam. We determined the minimum length of the QDM system to generate a periodic intensity profile with a visibility value equal to 1 for the probe field at the end of the medium. It is also shown that, by increasing the length of the QDM medium, the intensity profiles at the maximum areas become sharper. This feature was quantified by considering a sharpness factor for the intensity profile of the probe beam at the transverse plane. We also studied the resulting tunneling-induced Talbot effect. For this purpose, we presented the free space propagation of the induced periodic field and investigated the Talbot images of the produced periodic intensity patterns at the end of the QDM system under free space propagation. The presented adjustable method for the generation of periodic coherent intensity patterns might find many applications in science and technology. Especially in the optical lithography, by using the proposed method, the QDMs system can be used instead of nanofabricated physical transmission diffraction gratings. Based on the optical lithography applications of the presented method, an extension of the current research may be the production of two-dimensional arrays of intensity spots at the exit plane of a QDMs system.
